# Communicating Bad News in Emergency Health Care: Challenges and Best Practices – A Literature Review

**DOI:** 10.1192/j.eurpsy.2025.1860

**Published:** 2025-08-26

**Authors:** C. Pires-Lima, M. Figueiredo-Braga

**Affiliations:** 1 Neurosciences and Mental Health Department; 2 CINTESIS – Center for Health Technology and Services Research, Faculty of Medicine, University of Porto; 3 i3S – Institute for Research and Innovation in Health, University of Porto, Porto, Portugal

## Abstract

**Introduction:**

Communicating bad news (CBN) in emergency medicine is a crucial and challenging aspect of clinical care. Emergency Health Care (EHC) operates in high-pressure, time-sensitive environments, where life-altering information must be communicated rapidly, leaving little time for careful preparation or consideration. The emotional, ethical, and psychological complexities of CBN in such settings necessitate effective communication strategies to mitigate the distress of patients, families, and healthcare providers. This literature review aimed to examine the key challenges and best practices associated with delivering bad news in emergency medicine.

**Objectives:**

This review aims to synthesize the literature with the main objectives of 1. identifying the challenges faced by emergency medical professionals in delivering bad news and 2. collect the best practices, general guidelines, specific protocols and communication models used in emergency settings.

**Methods:**

A systematic review of the literature was conducted, focusing on peer-reviewed articles published between 2000 and 2023, using the keywords “comunicating bad news,” “emergency health care,” “communication in emergencies,” and “best practices for CBN.”. Articles were screened for relevance and rigor, and key findings were synthesized thematically.

**Results:**

The literature highlights several challenges: time constraints in EHC, lack of formal communication training, and emotional toll on both providers and recipients of the news. Emergency physicians often struggle to deliver bad news in a compassionate yet efficient manner, especially in the context of sudden or unexpected death. The best practices identified include the use of structured communication frameworks, such as the SPIKES protocol, which helps guide healthcare providers through the process. Multidisciplinary support, including involving social workers and counselors, was also emphasized as essential to alleviating the burden on both the physician and the patient. Furthermore, studies have revealed that when formal DBN training is implemented during residency, physician confidence and patient satisfaction improve significantly.

**Conclusions:**

The review underscores the complexity of delivering bad news in emergency medicine. Incorporating structured communication protocols and formal training into medical education and emergency department practices is essential for improving patient outcomes and physician well-being. Future research should focus on evaluating the effectiveness of tailored CBN protocols and programs as well as interdisciplinary collaboration.

**Disclosure of Interest:**

None Declared

